# Factors associated with underutilization of antenatal care services in Indonesia: results of Indonesia Demographic and Health Survey 2002/2003 and 2007

**DOI:** 10.1186/1471-2458-10-485

**Published:** 2010-08-16

**Authors:** Christiana R Titaley, Michael J Dibley, Christine L Roberts

**Affiliations:** 1 Sydney School of Public Health, Edward Ford Building (A27), University of Sydney, NSW 2006, Australia; 2 The Kolling Institute of Medical Research, Royal North Shore Hospital, University of Sydney, St Leonards, NSW 2065, NSW, Australia

## Abstract

**Background:**

Antenatal care aims to prevent maternal and perinatal mortality and morbidity. In Indonesia, at least four antenatal visits are recommended during pregnancy. However, this service has been underutilized. This study aimed to examine factors associated with underutilization of antenatal care services in Indonesia.

**Methods:**

We used data from Indonesia Demographic and Health Survey (IDHS) 2002/2003 and 2007. Information of 26,591 singleton live-born infants of the mothers' most recent birth within five years preceding each survey was examined. Twenty-three potential risk factors were identified and categorized into four main groups, external environment, predisposing, enabling, and need factors. Logistic regression models were used to examine the association between all potential risk factors and underutilization of antenatal services. The Population Attributable Risk (PAR) was calculated for selected significant factors associated with the outcome.

**Results:**

Factors strongly associated with underutilization of antenatal care services were infants from rural areas and from outer Java-Bali region, infants from low household wealth index and with low maternal education level, and high birth rank infants with short birth interval of less than two years. Other associated factors identified included mothers reporting distance to health facilities as a major problem, mothers less exposed to mass media, and mothers reporting no obstetric complications during pregnancy. The PAR showed that 55% of the total risks for underutilization of antenatal care services were attributable to the combined low household wealth index and low maternal education level.

**Conclusions:**

Strategies to increase the accessibility and availability of health care services are important particularly for communities in rural areas. Financial support that enables mothers from poor households to use health services will be beneficial. Health promotion programs targeting mothers with low education are vital to increase their awareness about the importance of antenatal services.

## Background

The proportion of child deaths occurring in the neonatal period-the first four weeks of an infants' life, has been increasing worldwide [1,2]. Antenatal care, a pregnancy-related service provided to pregnant women by health professionals, is among the major interventions which aim to prevent neonatal deaths and maintain the health of women during pregnancy [3,4]. A review on interventions for neonatal survival demonstrated that up to 12% of neonatal deaths could be averted by the provision of antenatal care services at 90% coverage [3].

Antenatal care enables health professionals to identify potential risks for the pregnancy or for the delivery and to provide prompt treatment for women experiencing health problems during pregnancy [4]. Through this service, women will receive assistance in developing a birth plan and be prepared for parenting after the childbirth [4]. Other services provided include the provision of Tetanus Toxoid vaccinations, iron/folic acid supplements and control of nutritional deficiencies [3,5].

Observational studies from both developed and developing countries have shown a protective effect of antenatal care services on neonatal deaths [6-10]. Inadequate antenatal care has been associated with adverse pregnancy outcomes [6,8,11]. Furthermore, studies from India have shown that women who attended antenatal care services had an increased likelihood of using trained delivery attendants during childbirth, or having an institutional delivery [12,13].

In Indonesia, at least four antenatal visits are recommended during pregnancy [14,15]. Antenatal care is provided by health personnel through different modes of service delivery, including the facility based and outreach services.

Descriptive data from the 2007 Indonesia Demographic and Health Survey (IDHS) [14] has shown that, as in other developing countries [16-19], antenatal care services in Indonesia are still underutilized. Approximately 95% of pregnant women in Indonesia attended at least one antenatal care visit; however only 66% of women had four antenatal visits as recommended, which is lower than the national target of 90% of women having at least four antenatal care visits [14]. The proportion of non-attendance at antenatal care services varied widely across provinces, ranging from 31% in Papua to less than 0.5% in DKI Jakarta [14]. This study therefore aimed to examine factors associated with underutilization of antenatal care services in Indonesia. The results should provide insights to policy makers about potential public health strategies to increase the uptake of antenatal care services.

## Methods

### Data sources

We used data from the Indonesia Demographic and Health Survey (IDHS) 2002/2003 and 2007, which are openly available from the Measure DHS website [20]. The IDHS is a five-year periodic survey used to collect information from ever-married women aged 15-49 years and ever-married men 15-54 years about demographic and health status. Three types of questionnaires used were the Household, Women's, and Men's Questionnaire [20]. The Women's questionnaire included questions about women's demographic characteristics, their reproductive history, pregnancy, postnatal care, as well as immunization and nutrition. The sampling method of the IDHS has been reported in detail elsewhere [21].

A total of 62,378 eligible women were interviewed for these two surveys; 29,483 in the 2002/2003 survey [22] and 32,895 women in the 2007 survey [14]. The response rate of eligible women in the 2002/2003 and 2007 IDHS was 98% [22] and 96% [14], respectively. In the present study, information of 26,591 singleton live-born infants of the mothers' most recent birth within five years preceding each survey was used. The most detailed health services information is available for a woman's most recent birth. Furthermore, this restriction aimed to reduce recall bias of mothers about their pregnancies.

### Variables

The primary outcome of this study was underutilization of antenatal care services which included infants whose mothers never attended antenatal care services and mothers attending less than the four recommended antenatal services [14,15]. Antenatal service referred to any pregnancy-related services provided by skilled health personnel, such as doctors, midwives, or village midwives. We excluded those antenatal care services provided by non-health professionals such as traditional birth attendants. A sensitivity analysis was also performed for infants whose mothers never attended any antenatal service for her last pregnancy within five years preceding each survey.

**Figure 1:**
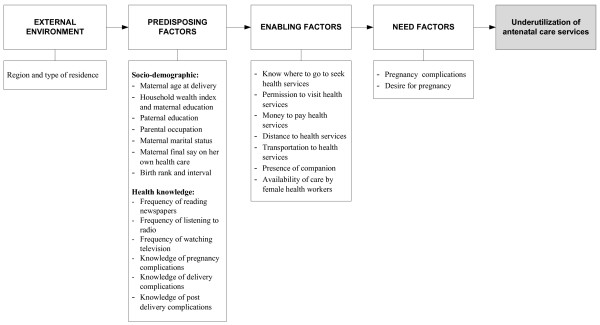
**Theoretical framework of factors associated with underutilization of antenatal care services in Indonesia**. Note: Framework adapted from Andersen behavioural model [23].

We adapted the behavioural model framework of Andersen [23] for use of health services, to group the factors potentially associated with not attending antenatal care services (Figure 1). Twenty-three potential risk factors were identified and categorized into four main groups, external environment, predisposing, enabling and need factors. The variables included in the study are presented in Figure 1.

A new household wealth index variable was constructed to rank households across the pooled IDHS data. Using a Principle Component Analysis method [24], weights were assigned to four housing characteristics (source of drinking water, type of toilet, main material of floor, main material of wall), and seven household assets (availability of electricity, possession of radio, television, fridge, bicycle, motorcycle, and car). A composite three-category variable of household wealth index, i.e. rich, middle and poor, and maternal education level, was constructed to assess their association with the outcome.

### Statistical analysis

Frequency tabulations were performed to describe the characteristics of infants included in this study. Logistic regression was used to determine factors associated with the outcome. Bivariate and multivariable analyses were conducted to assess the crude Odds Ratio (OR) and adjusted Odds Ratio (aOR), respectively.

Using a hierarchical modelling strategy [25], the more distal factors were first entered into the model to assess their associations with the study outcome. Backward elimination procedure was then employed to remove factors which were not significantly associated with underutilization of antenatal services, using a significance level of 0.05. A variable that represented year of survey was retained in all models regardless of its significance level.

In the first model, the year of survey variable and the external environment factor variable, i.e. the combined region and type of residence, were entered (see Figure 1). Backward elimination method was conducted to select factors significantly associated with the outcome. In the second model, thirteen predisposing factor variables were entered followed by backward elimination procedure. A similar approach was used for enabling and need factors. Interaction between maternal education and household wealth index was also examined.

All of the OR, aOR and 95% confidence interval (CI) were determined and weighted for the sampling probabilities. Statistical analyses performed in this study used the STATA/MP version 10.0 (2007) (Stata Corporation, College Station, TX, USA). Survey commands were employed for logistic regression models to adjust for the sampling weights and cluster sampling design.

The Population Attributable Risk (PAR) [26-28] was calculated for selected factors associated with the outcome to estimate the proportion of underutilization of antenatal care (ANC) services attributable to the factor examined. Using the adjusted odds ratio (aOR), the PAR was calculated as follows [26-28]:

$$\text{PAR}=\text{Proportion of infants whose mothers underutilized ANC associated with the factor}\frac{\;\left(\text{aOR}-\text{1}\right)}{\text{aOR}}$$

## Results

Of the 26,591 singleton live-born infants of the mothers' most recent birth within five years preceding each survey, 20% (95% CI: 18%-21%) were born to mothers attending less than four antenatal care services and this included 7% (95% CI: 7%-8%) of infants born to mothers who did not attend any services. The percentage of mothers attending less than four services decreased slightly from 20% in IDHS 2002/2003 to 19% in IDHS 2007.

Table 1 presents the baseline characteristics of mothers included in this analysis. It shows that compared to mothers from urban areas of the Java-Bali region, mothers from other areas were more likely to underutilize antenatal care services. Higher odds for not attending the services were found in rural than in urban areas.

**Table 1:** Baseline characteristics, unadjusted and adjusted odds ratio (OR) for factors associated with underutilization of antenatal care services in Indonesia, IDHS 2002/2003 and 2007

VARIABLE	N (%)*		UNADJUSTED^†^			ADJUSTED^†^		
			
			OR	(95% CI)	*P*	OR	(95% CI)	*P*
*Year of survey*								
IDHS 2002/2003 *(Ref)*	12646	(47.6)	1.00			1.00		
IDHS 2007	13945	(52.4)	0.93	(0.79-1.09)	0.37	0.87	(0.73-1.02)	0.09
								
**EXTERNAL ENVIRONMENT**								
*Region and type of residence*								
Java-Bali region: urban *(Ref)*	8101	(30.5)	1.00			1.00		
Java-Bali region: rural	7386	(27.8)	2.76	(2.04-3.75)	< 0.001	2.78	(2.05-3.76)	< 0.001
Sumatera region: urban	2031	(7.6)	1.63	(1.17-2.27)	< 0.01	1.63	(1.17-2.26)	< 0.01
Sumatera region: rural	3584	(13.5)	4.57	(3.47-6.03)	< 0.001	4.60	(3.49-6.07)	< 0.001
Eastern Indonesia region: urban	1645	(6.2)	2.39	(1.51-3.79)	< 0.001	2.39	(1.52-3.76)	< 0.001
Eastern Indonesia region: rural	3845	(14.5)	4.86	(3.76-6.29)	< 0.001	4.93	(3.82-6.36)	< 0.001
								
**PREDISPOSING FACTORS**								
**Socio-demographic**								
*Maternal age at delivery*	Mean ± SE = (27.2 ± 0.07)		1.01	(1.00-1.02)	< 0.01	0.97	(0.96-0.98)	< 0.001
								
*Household wealth index and maternal education*								
Wealthiest household, completed secondary education or higher *(Ref)*	5137	(19.3)	1.00			1.00		
Wealthiest household, completed primary or some secondary education	4273	(16.1)	3.47	(2.63-4.56)	< 0.001	2.15	(1.63-2.85)	< 0.001
Wealthiest household, incomplete primary education or none	548	(2.1)	9.45	(5.82-15.35)	< 0.001	4.01	(2.37-6.79)	< 0.001
Middle household wealth, completed secondary education or higher	1629	(6.1)	3.76	(2.72-5.21)	< 0.001	2.36	(1.67-3.32)	< 0.001
Middle household wealth, completed primary or some secondary education	5738	(21.6)	6.91	(5.31-9.00)	< 0.001	3.13	(2.30-4.25)	< 0.001
Middle household wealth, incomplete primary education or none	1584	(6.0)	17.86	(13.46-23.71)	< 0.001	5.50	(3.86-7.83)	< 0.001
Poorest household, completed secondary education or higher	425	(1.6)	10.24	(7.01-14.97)	< 0.001	4.30	(2.87-6.45)	< 0.001
Poorest household, completed primary or some secondary education	4059	(15.3)	14.58	(11.16-19.05)	< 0.001	4.75	(3.42-6.60)	< 0.001
Poorest household, incomplete primary education or none	2326	(8.7)	37.76	(28.32-50.35)	< 0.001	8.82	(6.10-12.73)	< 0.001
								
*Paternal education *								
Completed secondary education or higher *(Ref)*	9081	(34.2)	1.00			1.00		
Completed primary or some secondary education	13237	(49.8)	3.30	(2.87-3.79)	< 0.001	1.41	(1.19-1.66)	< 0.001
Incomplete primary education or none	4199	(15.8)	7.73	(6.52-9.18)	< 0.001	1.60	(1.31-1.95)	< 0.001
								
*Parental occupation*								
Unemployed mother and working father *(Ref)*	14100	(53.0)	1.00					
Working mother and working father	11820	(44.5)	1.19	(1.06-1.34)	< 0.01			
Unemployed father	581	(2.2)	1.71	(1.20-2.44)	< 0.01			
								
*Maternal marital status*								
Currently married *(Ref)*	25953	(97.6)	1.00			1.00		
Formerly married	638	(2.4)	1.85	(1.45-2.37)	< 0.001	1.60	(1.19-2.16)	< 0.01
								
*Maternal final say on her own health care*								
Woman with partner/other *(Ref)*	8514	(32.0)	1.00			1.00		
Woman alone	14200	(53.4)	1.09	(0.96-1.25)	0.19	1.23	(1.08-1.41)	< 0.01
Partner alone/someone else/other	3860	(14.5)	1.37	(1.17-1.61)	< 0.001	1.24	(1.05-1.47)	0.01
								
*Birth rank and interval*								
2^nd ^or 3^rd ^birth rank, > 2 year interval *(Ref)*	10751	(40.4)	1.00			1.00		
1^st ^birth rank	9139	(34.4)	0.86	(0.76-0.97)	0.01	0.81	(0.70-0.93)	< 0.01
2^nd ^or 3^rd ^birth rank, ≤ 2 year interval	1570	(5.9)	1.95	(1.61-2.37)	< 0.001	1.94	(1.58-2.40)	< 0.001
≥ 4^th ^birth rank, > 2 year interval	4443	(16.7)	2.67	(2.37-3.00)	< 0.001	2.08	(1.80-2.40)	< 0.001
≥ 4^th ^birth rank, ≤ 2 year interval	687	(2.6)	4.74	(3.74-6.00)	< 0.001	3.79	(2.88-4.99)	< 0.001
								
**Health knowledge**								
*Frequency of reading newspaper*								
At least once a week *(Ref)*	3773	(14.2)	1.00			1.00		
Less than once a week	9402	(35.4)	1.85	(1.52-2.26)	< 0.001	1.02	(0.82-1.27)	0.84
Never	13376	(50.3)	5.49	(4.50-6.71)	< 0.001	1.50	(1.20-1.86)	< 0.001
								
*Frequency of listening to radio*								
At least once a week *(Ref)*	8668	(32.6)	1.00					
Less than once a week	9369	(35.2)	1.22	(1.06-1.40)	0.01			
Never	8509	(32.0)	2.06	(1.77-2.39)	< 0.001			
								
*Frequency of watching television*								
At least once a week *(Ref)*	20634	(77.6)	1.00			1.00		
Less than once a week	3715	(14.0)	2.50	(2.17-2.89)	< 0.001	1.25	(1.07-1.46)	< 0.01
Never	2205	(8.3)	5.23	(4.35-6.28)	< 0.001	1.62	(1.34-1.94)	< 0.001
								
*Knowledge of pregnancy complications*								
Yes *(Ref)*	11867	(44.6)	1.00			1.00		
None	14717	(55.3)	2.73	(2.43-3.08)	< 0.001	1.46	(1.25-1.70)	< 0.001
								
*Knowledge of delivery complications*								
Yes *(Ref)*	13133	(49.4)	1.00			1.00		
None	13454	(50.6)	2.40	(2.15-2.68)	< 0.001	1.19	(1.04-1.36)	0.01
								
*Knowledge of post delivery complications*								
Yes *(Ref)*	8518	(32.0)	1.00					
None	18063	(67.9)	2.10	(1.87-2.37)	< 0.001			
								
**ENABLING FACTORS**								
*Know where to go to seek health services*								
Small problem *(Ref)*	25166	(94.6)	1.00					
Big problem	1403	(5.3)	2.24	(1.82-2.77)	< 0.001			
								
*Permission to visit health services*								
Small problem *(Ref)*	25352	(95.3)	1.00					
Big problem	1215	(4.6)	2.36	(1.93-2.88)	< 0.001			
								
*Money to pay health services*								
Small problem *(Ref)*	19662	(73.9)	1.00			1.00		
Big problem	6909	(26.0)	2.49	(2.21-2.80)	< 0.001	1.21	(1.05-1.39)	0.01
								
*Distance to health services*								
Small problem *(Ref)*	22580	(84.9)	1.00			1.00		
Big problem	3989	(15.0)	2.76	(2.39-3.18)	< 0.001	1.21	(1.03-1.42)	0.02
								
*Transportation to health services*								
Small problem *(Ref)*	22975	(86.4)	1.00					
Big problem	3593	(13.5)	2.91	(2.51-3.38)	< 0.001			
								
*Presence of companion*								
Small problem *(Ref)*	23688	(89.1)	1.00			1.00		
Big problem	2878	(10.8)	1.84	(1.58-2.14)	< 0.001	1.22	(1.01-1.47)	0.04
								
*Availability of care by female health workers*								
Small problem *(Ref)*	24338	(91.5)	1.00			1.00		
Big problem	2210	(8.3)	1.58	(1.31-1.90)	< 0.001	1.29	(1.04-1.59)	0.02
								
**NEED FACTORS**								
								
*Pregnancy complications*								
With complications *(Ref)*	2349	(8.8)	1.00			1.00		
No complications	24201	(91.0)	1.78	(1.46-2.17)	< 0.001	1.44	(1.16-1.78)	< 0.01
								
*Desire for pregnancy*								
Wanted then *(Ref)*	21560	(81.1)	1.00			1.00		
Wanted later	2907	(10.9)	1.46	(1.24-1.71)	< 0.001	1.38	(1.14-1.67)	< 0.01
Unwanted	2044	(7.7)	1.82	(1.52-2.17)	< 0.001	1.42	(1.13-1.78)	< 0.01

Note:

*) The total number varies between categories because of missing values

^†^) 1407 missing cases were excluded from the analysis

All values are weighted by the sampling probability

*Ref *: reference group

**Figure 2:**
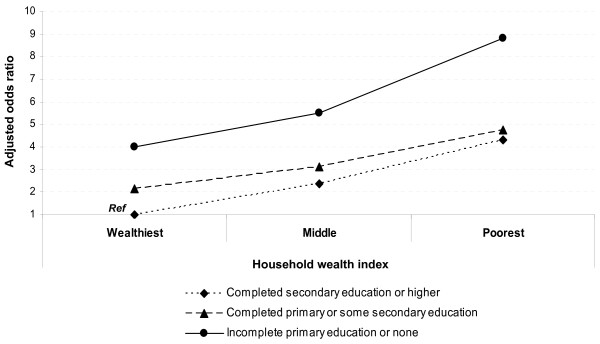
**Odds ratio of combined maternal education and household wealth index for underutilization of antenatal care services**. Note: a. Ref: reference group. b. Model adjusted for year of survey, region and type of resident, maternal age at delivery, paternal education, maternal marital status, maternal final say on her own health care, birth rank and interval, maternal frequency of reading newspaper, maternal frequency of watching television, maternal knowledge of pregnancy complications and maternal knowledge of delivery complications.

Among the socio-demographic factors, the odds for underutilizing antenatal care services increased significantly for mothers with low educational attainment and from households with a low wealth index. A significant interaction term was found between household wealth index and maternal education (p = 0.02) (Figure 2). The association between household wealth index and underutilization of antenatal care services was influenced by maternal education level. An increased education level has a greater effect for women from households with a low wealth index compared to those from the wealthiest households (Table 1 and Figure 2).

Another significant demographic factor identified was the combined birth rank and interval. Mothers of high birth rank infants with a short birth interval were more likely to underutilize antenatal services. However, a reduced odds was observed for first birth rank infants (aOR = 0.81, 95% CI: 0.70-0.93), compared to the second and the third birth ranked infants with more than two-year birth interval. Other factors significantly associated with underutilization of antenatal services were women with less exposure to mass media, women lacking knowledge about obstetric complications, women reporting physical distance to health facilities as a major problem, women who reported they did not experience any pregnancy complications and women who did not intend to become pregnant at the time of pregnancy.

Similar findings were found for mothers who did not attend any antenatal services (data not shown). An increased odds of not attending antenatal care services was found among women from the outer Java-Bali region and from rural areas, women from poor households and with a low level of education, women lacking knowledge of pregnancy complications (aOR = 1.46, 95% CI: 1.25-1.70, p < 0.001), women who reported money to pay health services as a major problem (aOR = 1.21, 95% CI: 1.05-1.39, p = 0.01), and women who did not have any pregnancy complications (aOR = 1.44, 95% CI:1.16-1.78, p < 0.01).

Table 2 presents the PAR for selected factors associated with underutilization of antenatal care. The combined PAR of household wealth and maternal education showed that in the population 55% of the total risk for underutilizing antenatal care services was attributable these two variables. The PAR increased along with the reduction of household wealth index and maternal education level. The PAR values presented in Table 2 should not be added, as this might cause misinterpretation of the results [29].

**Table 2:** Adjusted Population Attributable Risk (PAR) for selected risk factors, IDHS 2002/2003 and 2007*

VARIABLE	%^†^	aOR^‡^	PAR (95%CI)	
**Region and type of residence**				
Java-Bali region: urban *(Ref)*	0.14	1.00		
Java-Bali region: rural	0.30	2.78	0.19	(0.13 0.25)
Sumatera region: urban	0.05	1.63	0.02	(0.01 0.04)
Sumatera region: rural	0.21	4.60	0.16	(0.13 0.20)
Eastern Indonesia region: urban	0.06	2.39	0.03	(0.01 0.07)
Eastern Indonesia region: rural	0.24	4.93	0.19	(0.16 0.22)

*Combined risk *			*0.48*	*(0.38 0.58)*

**Household wealth index and maternal education**				
Wealthiest household, completed secondary education or higher *(Ref)*	0.03	1.00		
Wealthiest household, completed primary or some secondary education	0.08	2.15	0.04	(0.03 0.06)
Wealthiest household, incomplete primary education or none	0.02	4.01	0.02	(0.01 0.03)
Middle household wealth, completed secondary education or higher	0.03	2.36	0.02	(0.01 0.03)
Middle household wealth, completed primary or some secondary education	0.20	3.13	0.14	(0.10 0.17)
Middle household wealth, incomplete primary education or none	0.11	5.50	0.09	(0.07 0.11)
Poorest household, completed secondary education or higher	0.02	4.30	0.02	(0.01 0.02)
Poorest household, completed primary or some secondary education	0.25	4.75	0.20	(0.16 0.23)
Poorest household, incomplete primary education or none	0.25	8.82	0.22	(0.18 0.25)

*Combined risk*			*0.55*	*(0.46 0.63)*

**Birth rank and interval**				
2^nd ^or 3^rd ^birth rank, > 2 year interval *(Ref)*	0.33	1.00		
1^st ^birth rank	0.25	0.81		
2^nd ^or 3^rd ^birth rank, ≤ 2 year interval	0.08	1.94	0.04	(0.03 0.05)
≥ 4^th ^birth rank, > 2 year interval	0.28	2.08	0.15	(0.12 0.18)
≥ 4^th ^birth rank, ≤ 2 year interval	0.06	3.79	0.05	(0.03 0.06)

*Combined risk*			*0.22*	*(0.17 0.27)*

**Knowledge of pregnancy complications**				
Yes *(Ref)*	0.26	1.00		
None	0.74	1.46	0.23	(0.14 0.31)
				
**Distance to health services**				
Small problem *(Ref)*	0.73	1.00		
Big problem	0.27	1.21	0.05	(0.01 0.09)

Note:

*) These PAR values should not be added. If necessary, only the lower limit of the PAR could be added to provide conservative estimate about the potential prevention due to the elimination of multiple risk factors [29];

^†^) Weighted by the sampling probability

^‡^) aOR = adjusted odds ratio

*Ref *: reference group

## Discussion

### Main findings

Our study demonstrated that factors strongly associated with underutilization of antenatal care services were region and type of residence, community's household wealth index and maternal education and combined birth rank and birth interval. Mothers of infants from the outer Java-Bali region, particularly from rural areas were more likely to underutilize antenatal services. A significant interaction between household wealth index and maternal education was also found. Low economic status was associated with increased odds of underutilizing antenatal care services among mother with a low education level. Furthermore, an increased odds of underutilizing antenatal care services was observed in mothers of high birth rank infants. Other significant factors found included mothers less exposed to mass media, mothers reporting money to pay for health services as a major problem and mothers reporting distance to health facilities as a major problem. Amongst the need factors, the odds for underutilizing antenatal services increased among mothers who did not experience any pregnancy complications. The identification of these factors is important to develop public health strategies that address key issues which hinder women from utilizing antenatal service in Indonesia.

### Factors associated with underutilization of antenatal care services

Outer Java-Bali region and rural residence were associated with underutilization of antenatal services. This is likely due to the shortage of health services aggravated by the limited access in outer islands, especially in rural areas. A study in Java [30] reported a shortage of health care providers which was reflected by a low density of health professionals compared to the international standard [31]. In this present study, the problem of access to services was confirmed by the increased likelihood for underutilization of antenatal services among mothers reporting distance to health services as a major problem. A previous study has shown that distance and time to the nearest health facilities influenced health services utilization [32]. A qualitative study from West Java Province found that in rural areas, a long travel time worsened by poor road conditions prevented communities from attending antenatal services [33]. These findings indicate that the improvement of access to health services as well as the distribution of health services and personnel, especially in rural areas, should be a priority.

The role of household economic status on health services utilization has been reported in various studies [34,35]. An increased likelihood to underutilize antenatal services along with the reduction of household wealth index was also found in our study. Women from high household wealth index were more likely to be able to afford health services, and their associated costs, including transportation costs [16]. Although the Health Insurance Scheme for the Population or *Jaminan Kesehatan Masyarakat (Jamkesmas)*, which is an insurance scheme provided to the poor or near poor communities in Indonesia to give them free access to health services has been implemented, our earlier study showed that it did not improve the health seeking behaviour of some poor communities, particularly those living in rural and remote areas [33]. This might be due to the lack of understanding of *Jamkesmas *and how it can be used by some community members. Urgent attention to implement effective communication programs to support appropriate use of this insurance scheme is required, along with evaluation and monitoring strategies to assess its effectiveness in reaching the target population. Efforts to strengthen community financing mechanisms might also help women from low economic status households to access health services [36].

Low household economic status is correlated with low education level [37]. This study demonstrated a strong association between low maternal education and underutilization of antenatal service. The relationship between maternal education and health service utilization was also reflected by increased odds among mothers with lack of knowledge of obstetric complications and lack of exposure to mass media, as reported in previous literature [18,38]. Moreover, our study confirmed that lack of knowledge about the importance of maternal and child health hinders women from attending antenatal services. Women reporting not having any pregnancy complications had an increased odds of underutilizing antenatal care services. This might be because they felt well during pregnancy and therefore did not perceive the need to attend any services. Several pathways have been suggested through which maternal education might affect health care utilization, including greater knowledge of the importance of health services among highly educated women and the increased ability to select the most appropriate service for their needs [37,39]. As reflected by the high PAR of combined low household wealth index and low maternal education status, health promotion programs targeting low educated mothers from financially deprived households about the importance of antenatal services will be beneficial to increase the uptake of these services.

Similar to our findings, the association between high parity and low utilization of health services has been reported in another study from Turkey [40]. Women with high parity might tend to rely on their experiences from previous pregnancies and not feel the need for antenatal checks [18,40]. Some might experience difficulties to attend antenatal services due to time constraints related to their responsibilities for their other children [18,34,40].

Other factors associated with antenatal care utilization found in our study included maternal desire for pregnancy and women's autonomy. Women with an unwanted pregnancy are more likely to underutilize antenatal services [41]. Unwanted pregnancies are associated with late start or less frequent antenatal visits compared to wanted pregnancies [16,41]. Moreover, our study showed that women who were not involved in the final decision making about their own health care were more likely to underutilize antenatal services. An increased likelihood for underutilizing antenatal services was found in women who were the only decision maker about their own health care, compared to those who involved others in the decision making. This finding showed the vital role of family support in utilizing health services. It also indicates that public health strategies should target not only the pregnant women but also other family members to increase their awareness about the importance of antenatal services.

### Strengths and limitations

This present study was based on large representative national surveys, the 2002/2003 and 2007 IDHS. The potential of recall bias has been minimized by restricting the sample only to mothers' most recent delivery within the last five years of each survey. The large sample used in this study allows the examination of various potential risk factors, the external environment, predisposing, enabling, and need factors. This also increased the validity of the study results. The following limitations of our study should be kept in mind when interpreting the results. As with other cross-sectional survey data, the interpretation of the causality of factors associated with underutilization of antenatal care is restricted by the study design. The information used is also subject to recall bias, as information collected relied on the women's recall ability about her pregnancy. The selection of potential risk factors was driven by the availability of information in each IDHS. Furthermore, some variables were not infant-specific, such as parental occupation, since they informed the employment status of parents within one year prior to the survey. However, these limitations are unlikely to impact on the validity of the analyses.

## Conclusions

Our study found significant associations between external environment, predisposing, enabling and need factors and underutilization of antenatal care services. Strategies to increase the accessibility and availability of health care services should be a priority in Indonesia, particularly in rural areas. As reflected by the PAR results, it is important for health promotion programs to target women with low levels of education and from poor households to increase their awareness about the importance of antenatal services and to increase their uptake of these services. Financial support that enables mothers from poor households to use health services will be beneficial to reduce their out-of-pocket expenditure for both medical and transportation costs. Evaluation and monitoring strategies of the current financial supports are important to assess their effectiveness in reaching the target population.

## Competing interests

The authors declare that they have no competing interests.

## Authors' contributions

CRT and MJD participated in the design of the study. CRT performed the analysis and prepared the manuscript. MJD and CLR provided data analysis advice and revision of the manuscript. All authors read and approved the final manuscript.

## Pre-publication history

The pre-publication history for this paper can be accessed here:

http://www.biomedcentral.com/1471-2458/10/485/prepub
